# Age is just a number – and so is frailty: Strategies to inform resource allocation during the COVID-19 pandemic

**DOI:** 10.1017/cem.2020.358

**Published:** 2020-04-01

**Authors:** Kevin F. Boreskie, Patrick E. Boreskie, Don Melady

**Affiliations:** * Faculty of Applied Health Sciences, University of Manitoba, Winnipeg, MB; † Max Rady College of Medicine, Rady Faculty of Health Sciences, University of Manitoba, Winnipeg, MB; ‡ Department of Emergency Medicine, Max Rady College of Medicine, Rady Faculty of Health Sciences, University of Manitoba, Winnipeg, MB; § Don Melady, Schwartz-Reisman Emergency Medicine Institute, Department of Family and Community Medicine, University of Toronto, Toronto, ON

**Keywords:** Coronavirus disease 2019 (COVID-19), frailty, SARS-CoV-2, triage

As cases of critical COVID-19 patients have taxed their resources, hospitals in China and Europe have faced the difficult task of establishing criteria for which patients receive which level of care. Hospitals in Italy during this pandemic seem overwhelmed, leaving physicians with little guidance on how to triage patients and allocate therapeutic resources.^1^ Based on current rates of critical care admission in China and Italy, in a worst-case scenario, Canada would have a deficit of thousands of intensive care unit (ICU) beds in the peak of a national epidemic—a problem that will disproportionately affect older adults. This highlights the necessity of sound geriatric principles in the emergency department (ED) that incorporate the essential concept of frailty. We propose that a structured, evidence-based assessment of frailty, and not just noting the person's age, will help guide ED care during the COVID-19 pandemic.

When the Italian College of Anesthesia, Analgesia, Resuscitation, and Intensive Care (SIAARTI) released guidelines for the triaging of limited resources, they were criticized as being “age-ist.” Although not based entirely on chronological age, they suggested that an age cutoff for ICU care may ultimately be needed.^1^ This was especially interesting in Italy, a country with one of the oldest populations in the world. Although they explicitly recognize that age should not be the only consideration, these recommendations seem to propagate the misguided notion that being elderly and being frail are synonymous. We speculate that the decision was based on an observation that patients presenting with comorbidities, such as diabetes, cardiovascular disease, and immunocompromising conditions, have higher COVID-19 mortality.^2^ However, the correlation between polymorbidity and age is far from perfect, due to the heterogeneity of aging.^3^

As this pandemic develops, emergency physicians must be familiar with a more global approach to the assessment of patients’ physiologic resilience and chance of benefit with intensive care. This approach may seem more philosophical than practical in this crisis, but it can be an efficient tool to guide equitable resource allocation. This may put us closer to “getting it right” with medical care that makes a difference and maximizes benefit.^4^

Faced with fewer ICU beds per capita, physicians in the United Kingdom (UK) have been challenged with the same difficult resource allocation decisions as Italy. The proposed method of triage published by the National Institute for Health and Care Excellence (NICE) in the United Kingdom takes a slightly different approach. Instead of making decisions based on chronological age, the NICE *COVID-19 Rapid Guideline: Critical Care* proposes screening based on frailty for decisions regarding level of care.^5^ Frailty is characterized by widespread physiological declines across many systems that can leave a person more susceptible to health stressors, such as infection.^6^ Frail individuals are also more likely to present with a variety of comorbidities that each contribute to poor outcomes. While frailty is often associated with older age, purely age-based criteria are frequently inaccurate, as younger people can also present with frailty, and many older people are healthy and robust.^7^ Assessing frailty is already an unconscious part of an emergency physician's assessment of patients, but can be done in a more structured and evidence-based way.

**Figure 1.**
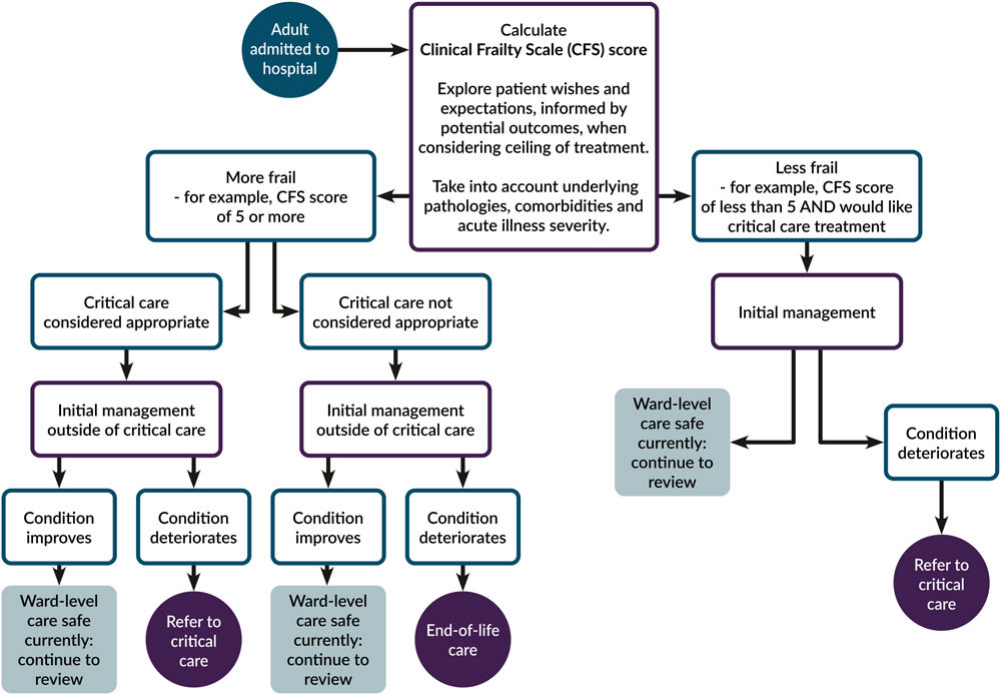
NICE flow chart for critical care management decisions during the COVID-19 pandemic.^5^

There are many methods of assessing frailty and no one method is universally accepted. Strategies for identifying frailty are often based on a balance between efficacy and feasibility in a specific setting. The COVID-19 guideline presented by NICE proposes the use of the Clinical Frailty Scale (CFS).^8^ The CFS, developed by Canadian geriatrician Kenneth Rockwood, is a simple one-page “frailty cheat sheet,” which provides nine pictograms and brief descriptions to place patients on a scale from 1 (very fit) to 9 (terminally ill). Patient placement on this scale is based on evaluation by an experienced clinician using a summary assessment of health and recent function to estimate their level of frailty or robustness. This tool has been validated and successfully used in multiple settings, including those where more extensive testing is not feasible.^9^ It is a rapid and ED-friendly method already used in every National Health Service (NHS) ED in the United Kingdom, and seems practical to use in the context of COVID-19 patients presenting to an overwhelmed hospital (Figure 1).

Most clinicians are aware that over the next few weeks, difficult decisions will need to be made around access to care for all patients, especially for those at greater risk of morbidity and mortality from COVID-19. The NICE COVID-19 guidelines could be used to provide a sensible decision support tool that integrates sound geriatric principles into the daily decision-making of emergency physicians: (1) *Make a holistic assessment of frailty.* This is simplified for contexts such as the ED by the validated Clinical Frailty Scale. (2) *Consider the risk-benefit for* this *patient.* Avoid basing treatment on evidence from studies of patients who are *not* like this patient. (3) *Discuss goals of care.* Frame the conversation around “what the person wants” and not “what we can do,” informed by an honest disclosure of prognosis.

While the NICE tool is specifically intended for use after the decision to admit, we suggest it could be modified to inform the decision of whether to admit, or even for prehospital disposition decisions regarding different levels of health center if frailty category was already known. These are hard decisions, which sometimes have been deferred to “others” (internists, intensivists, palliative care) but may soon be an ED decision as the demand on limited health care resources increases. Considering the Canadian context lacks the UK's NHS “4-hour standard” for admission decision, it can be assumed that many of the clinical decision points described in the NICE pathway may occur while under the care of emergency physicians.

As COVID-19 case incidence increases, Canada may be forced to make the tough decisions that other countries have already had to face, as anticipated numbers of patients presenting in critical condition will exceed the supply of available beds and ventilators. Faced with this difficulty, Canadian physicians may consider using frailty assessments as a means of improving care in this pandemic scenario. Research should prioritize studying outcomes of older patients and frail patients with COVID-19 to better characterize these populations and inform decision-making. In these uncertain times, we need to realize that it is likely not age, but rather frailty, that best contributes to outcome prediction. After all, the more encouraging interpretation of the mortality statistics in Italy for COVID-19 patients over 80 years of age is that over 80% are surviving.^10^
